# Dihydroartemisinin Alleviates the Symptoms of a Mouse Model of Systemic Lupus Erythematosus Through Regulating Splenic T/B-Cell Heterogeneity

**DOI:** 10.3390/cimb47070528

**Published:** 2025-07-09

**Authors:** Haihong Qin, Xiaohua Zhu, Xiao Liu, Yilun Wang, Jun Liang, Hao Wu, Jinfeng Wu

**Affiliations:** 1 Department of Dermatology, Huashan Hospital, Fudan University, Shanghai 200040, China; b0654@huashan.org.cn (H.Q.); xiaohuazhu@fudan.edu.cn (X.Z.); yolendawang@163.com (Y.W.); liangjun1976@medmail.com.cn (J.L.); 2 The Second Affiliated Hospital, Yunnan University of Chinese Medicine, Kunming 650041, China

**Keywords:** lupus, dihydroartemisinin, spleen cells, single-cell RNA sequencing

## Abstract

Background**:** Systemic lupus erythematosus (SLE) is a complex autoimmune disease with significant therapeutic challenges. Recent studies suggest that dihydroartemisinin (DHA), a traditional Chinese medicine known for its anti-malarial properties, may be beneficial for SLE treatment, although its precise mechanism remains unclear. This study aimed to investigate the effects of DHA on the cellular composition and molecular events of splenic T cells and B cells in MRL/lpr mice, a widely used SLE model. Methods: T cells and B cells isolated from the spleens of three DHA-treated mice and three control mice underwent single-cell RNA sequencing (scRNA-seq) using the 10× Genomics Chromium system. Comprehensive analyses included cell clustering, signaling pathway enrichment, pseudotime trajectory analysis, and cellular communication assessment using unbiased computational methods. Results: DHA treatment significantly reduced kidney inflammation and altered the proportions of splenic T cells and B cells, particularly decreasing plasma cells. Molecular profiling of effector CD4+ T cells showed a significant reduction in several inflammation-related signaling pathways in DHA-treated mice. Cellular communication analysis indicated altered interactions between effector CD4+ T cells and B cells in MRL/lpr mice after DHA treatment. Conclusions: Our findings reveal changes in cellular composition and signaling pathways in splenic T cells and B cells of MRL/lpr mice following DHA treatment. DHA may inhibit B-cell differentiation into plasma cells by modulating effector CD4+ T cells, potentially through the regulation of HIF1α and ligand–receptor interactions, enhancing our understanding of DHA’s mechanisms in SLE treatment.

## 1. Introduction

Systemic lupus erythematosus (SLE) is a chronic autoimmune disease characterized by recurrent inflammation and damage to multiple organs, including the kidneys, skin, and brain [1]. Conventional treatments such as glucocorticoids, immunosuppressive agents, nonsteroidal anti-inflammatory drugs, and hydroxychloroquine have shown variable efficacy and can lead to severe adverse effects [2,3]. Emerging biological targeted medicines like rituximab and belimumab show promise, but their efficacy has not yet surpassed that of traditional medications, with approximately 32% of patients experiencing adverse reactions such as infections [4,5]. This underscores the complex and heterogeneous nature of SLE, presenting a global therapeutic challenge.

With advancements in traditional Chinese medicine, there is increasing interest in its potential role in treating immune-related diseases, including SLE [6,7]. Dihydroartemisinin (DHA), a water-soluble metabolite of the Chinese herbal medicine artemisinin, has demonstrated notable anti-malarial and anti-tumor effects [8,9,10,11]. Recent studies suggest its potential in SLE treatment, highlighting its ability to inhibit the production of anti-dsDNA antibodies and improve pathological lesions of lupus nephritis in animal models [12,13]. Additionally, DHA has been found to suppress LPS-induced cell activation by inhibiting the TLR4/IRF/IFN pathway and reversing the senescence of myeloid-derived suppressor cells (MDSCs) through the Nrf2/HO-1 pathway, thus attenuating SLE development [14,15]. However, the precise mechanism of DHA in SLE treatment remains incompletely understood.

SLE is primarily mediated by the production of various autoantibodies against self-antigens and immune complexes. Although the exact pathogenic mechanisms are not fully elucidated, accumulating evidence suggests that dysfunctional T cells, particularly CD4+ T cells, play a crucial role in activating B cells and promoting autoantibody production [16,17]. The spleen, a primary peripheral lymphoid organ, is pivotal in immune regulation and B-cell antibody production. Dysregulation of splenic function is closely associated with SLE pathogenesis. While some studies have provided insights into the role of DHA in regulating splenic T-cell function in a *P. berghei* ANKA-infected female BALB/c mouse model [18], its impact on cell composition and underlying molecular events in the spleen during lupus remains unclear.

This study aimed to further investigate the mechanism of DHA in SLE therapy. T cells and B cells were isolated from the spleens of MRL/lpr mice using magnetic-activated cell sorting (MACS) and analyzed using single-cell RNA sequencing (scRNA-seq) with the 10× Genomics Chromium system. Our results indicate that DHA alters the composition and communication of splenic T and B cells, leading to the attenuation of lupus symptoms in mice.

## 2. Materials and Methods

### 2.1. Experimental Animals

Female MRL/lpr mice aged 4–6 weeks were purchased from the Shanghai Laboratory Animal Center (Shanghai, China) and housed under pathogen-free conditions. All experimental procedures adhered to animal husbandry guidelines. Ethical approval for the animal study was obtained from the Independent Ethics Committee of Huashan Hospital (No.: 2019 Huashan Hospital JS-098).

### 2.2. Dihydroartemisinin Treatment

The mice were randomly divided into two groups: the dihydroartemisinin (DHA)-treated group (DM group) and the saline solution-treated group (Control, M group), with 10 mice per group. Urinary protein levels (Catalog no: RE3997-96T, Bioruyee, Beijing, China) were monitored weekly. Mice with protein levels exceeding 30 mg/dL received daily intragastric administration of either DHA (100 mg/kg, Shanghai Winherb Company, Shanghai, China) or saline solution (Shanghai Changzheng Fumin Jinshan Pharmacological Company, Shanghai, China) for 12 weeks. At the end of the treatment period, three mice from each group were randomly selected and euthanized by cervical dislocation. Subsequently, spleen, kidney, and skin tissues were collected for further analysis.

### 2.3. Tissue Histological Study

Spleen, kidney, and skin tissues were fixed in 4% paraformaldehyde overnight, embedded in paraffin, and sectioned at a thickness of 5 μm. Standard hematoxylin and eosin (HE) staining was performed, and images were captured and processed using a digital slide scanner (Pannoramic MIDI, 3DHISTECH, Budapest, Hungary) and analyzed with Caseviewer software 2.4 (Servicebio, Wuhan, China).

### 2.4. Isolation of Splenic T and B Cells

Spleens were collected and pooled from three DHA-treated mice and three control mice, respectively. They were then gently mashed with a syringe plunger and filtered through a 70 μm mesh (BD Biosciences, Franklin Lakes, NJ, USA). After centrifugation at 400× *g* for 5 min, red blood cells were lysed using RBC Lysis Solution (Beyotime, Shanghai, China). For T-cell isolation, freshly isolated splenic cells were incubated with anti-CD3 magnetic beads (Catalog no. 130-094-973, Miltenyi Biotec, Cologne, Germany) for 15 min at 4 °C in the dark and sorted using MACS separators (Miltenyi Biotec, Cologne, Germany). Similarly, B cells were isolated using anti-CD19 magnetic beads (Catalog no. 130-121-301, Miltenyi Biotec, Cologne, Germany).

### 2.5. Single-Cell RNA Sequencing

Isolated T cells or B cells were resuspended in PBS with 2% fetal bovine serum (FBS). Cell counts and viability were determined using Countstar (Aber Instruments Ltd., Aberystwyth, UK). Single-cell RNA sequencing was performed following the manufacturer’s instructions for the Single Cell 3’ Library and Gel Bead Kit V3 and Chromium Single-Cell B Chip Kit (10× Genomics, performed by CapitalBio, Beijing, China). This process included GEM (gel bead-in-emulsion) generation, barcoding, cDNA amplification, library construction, and sequencing. Libraries were sequenced using an Illumina NovaSeq6000 sequencer(Illumina, Inc., San Diego, CA, USA) with a sequencing depth of at least 100,000 reads per cell and 150 bp (PE150) paired-end reads (performed by CapitalBio, Beijing, China).

### 2.6. scRNA-Seq Data Processing and Quality Control

Raw sequencing data were filtered and mapped to GRCm38 using Cell Ranger Version 3.1.0. Cell doublets were identified and removed using Scrublet. The Seurat package in R was used for single-cell RNA sequencing analysis. Quality control removed low-quality cells with fewer than 200 detected genes or mitochondrial gene expression exceeding 25%. Variable genes were identified using the FindVariableFeatures function, and the top 2000 highly variable genes were selected for principal component analysis (PCA). Batch effects among different groups were corrected using the Harmony package. The ElbowPlot function was used to identify significant PCs, with the first 40 PCs used for t-distributed stochastic neighbor embedding (t-SNE) clustering analysis in Seurat (resolution = 0.6). DEGs in each cluster were determined using the FindAllMarkers function, comparing each cluster with all other clusters. Average gene expression for each cluster or cell type was calculated using the AverageExpression function. t-SNE plots visualize single-cell distribution across clusters, sample groups, or individual samples. Gene expression levels were further visualized using feature plots, stacked violin plots, and dot plots. Additional R packages, such as pheatmap (version 1.0.12), ggplot2 (version 1.0.12), dplyr (version 1.1.4), and RColorBrewer (version 1.1.3), were used for stacked bar plots and heatmaps. Sub-clustering analysis followed the same steps, with cells involved in the second clustering analysis of a cell type removed to minimize sequencing bias.

### 2.7. Pseudotime Trajectory Analysis

R package Monocle 2 (version 2.30.0) was used to map potential differentiation associations among expanded subclusters of cell types. A new cell dataset object was constructed from cluster-annotated Seurat objects using the new cell dataset function. Differential gene tests were performed to identify differentially expressed genes (DEGs) for each cluster. Genes with a q value < 1 × 10^−15^ were used for pseudotime sorting, with only the top 2000 variable genes analyzed. Dimensionality reduction was achieved using the Discriminative Dimensionality Reduction via Learning a Tree (DDRTree) algorithm, and cells were sorted along the trajectory. The Branch Expression Analysis Model (BEAM) identified genes with branch-dependent expression. Further functional assays using Monocle 2’s plot_genes_branched_heatmap revealed key genes involved in the differentiation process.

### 2.8. Gene Set Enrichment Analysis (GSEA)

Gene set enrichment analysis (GSEA) utilized gene sets from the Molecular Signatures Database (MSigDB) to explore differentially enriched biological pathways between clusters or groups. GSEA was conducted using several R packages, including dplyr (version 1.1.4), msigdbr (version 7.5.1), clusterProfiler (version 4.10.0), and org.Mm.eg.db (version 3.18.0). A normalized enrichment score (NES) was calculated, considering differences in pathway size (i.e., gene set size), allowing for comparisons between pathways within the analysis. The NES reflects the degree to which a gene set is overrepresented at the top or bottom of a ranked list of genes, with positive and negative NES values representing enrichment at the top and bottom of the list, respectively. The analysis involved 1000 gene set permutations, with gene sets limited to 10–500 genes. Initially, a q value of 25% was used for all bioinformatics analyses.

### 2.9. Cell–Cell Communication Analysis with CellChat

Cell-to-cell communication analysis of potential receptor–ligand pairings was performed using the R package CellChat (version 1.6.1). Gene expression levels were calculated relative to the total read mapping to the same set of coding genes across all transcriptomes. Expression values were averaged within each single-cell cluster or cell sample. Visualizations were generated using pheatmap (version 1.12) and ggplot2 (version 1.0.12).

### 2.10. Statistical Analysis

Biologically significant differences were determined using default algorithms implemented in R packages.

## 3. Results

### 3.1. DHA Significantly Attenuated Clinical Symptoms and Renal Inflammation of MRL/lpr Mice

Our results showed a significant reduction in spleen volume in DHA-treated mice compared to controls. A noticeable reduction in hair loss was also observed in DHA-treated mice.

**Figure 1:**
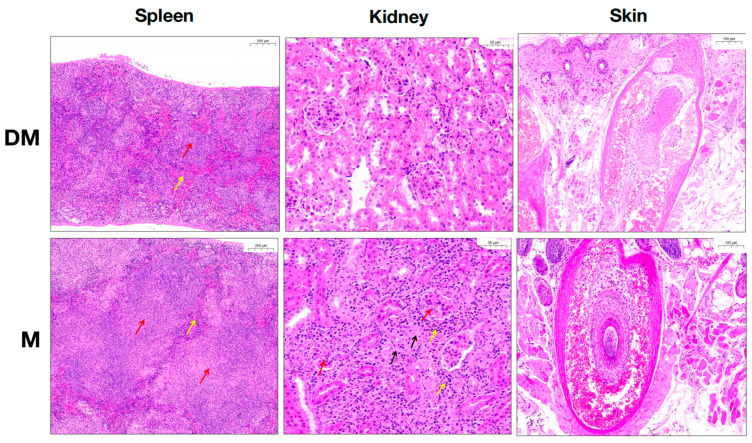
Pathogenic changes in spleen, kidney, and skin tissue between DHA-treated and control mice. HE staining was used to observe changes in spleen, kidney, and skin in both DHA-treated and control mice. Red arrow in spleen: white pulp; yellow arrow in spleen: red pulp; red arrow in kidney: renal glomeruli; yellow arrow in kidney: inflammatory cell infiltration; black arrow in kidney: interstitial fibrous tissue proliferation. Scale bars: 50 μm (spleen), 100 μm (skin), 200 μm (kidney). Data are representative of 3 independent experiments. DM: DHA-treated mice; M: control mice.

To further confirm DHA’s effect on lupus mice, we performed histopathological analysis on spleen, kidney, and skin sections. As shown in Figure 1, the white pulp was markedly decreased while the red pulp was increased in the spleen of DHA-treated mice. Additionally, atrophy of renal glomeruli, inflammatory cell infiltration, and interstitial fibrous tissue proliferation in the kidney were significantly reduced in the DHA-treated group. No differences were observed in skin tissue between the two groups, possibly because the areas of hair loss may not be the regions where the most significant pathological changes occur. Additionally, DHA may suppress some immune responses, but due to the complexity of skin tissue, it might not have significantly affected the immune cell composition and tissue pathology in the short term. These results collectively demonstrate that DHA treatment alleviates lupus manifestations, particularly renal inflammation.

### 3.2. scRNA-Seq Workflow and Clustering of Sorted T Cells and B Cells from Spleen

Numerous studies have indicated that abnormal spleen immunity contributes to lupus pathogenesis. Here, we assessed splenic T- and B-cell heterogeneity in MRL/lpr mice with or without DHA treatment using scRNA-seq.

**Figure 2:**
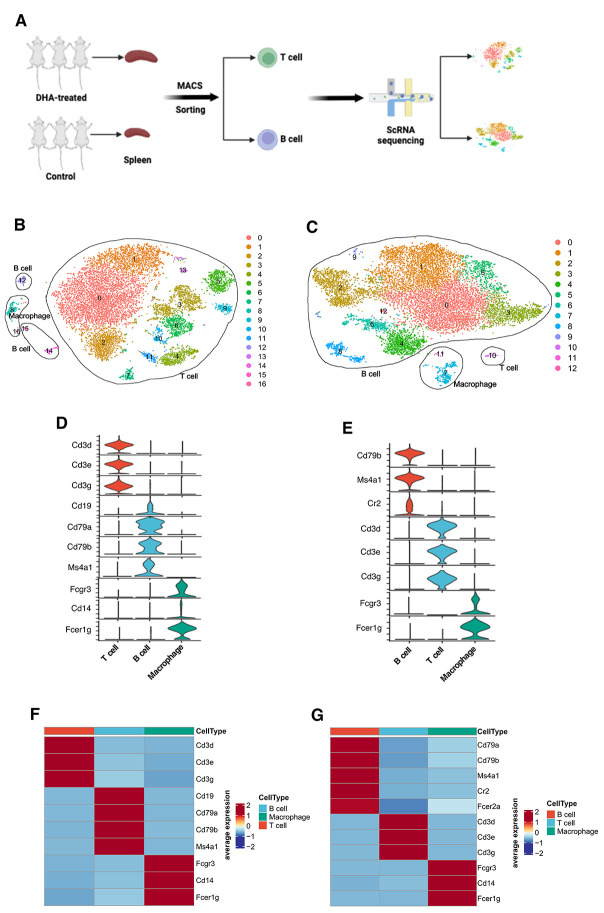
scRNA-seq analysis of isolated T and B cells from the spleen using MACS sorting in both DHA-treated and control mice. (**A**): Schematic workflow of sample preparation, MACS sorting, sequencing, and bioinformatics analysis. (**B**,**C**): t-SNE visualization of main cell types in all sorted T cells (*n* = 11,651) with 16 clusters (**B**) and in all sorted B cells (*n* = 13,481) with 12 clusters (**C**). Each dot represents a cell, colored according to its cluster. (**D**,**E**): Canonical marker gene expression for each cell type of sorted T cells (**D**) and sorted B cells (**E**) shown using stacked violin plots. The violin chart is colored by cell classification, with height representing gene expression levels and width representing the ratio of gene expression. (**F**,**G**): Canonical marker gene expression for each cell type of sorted T cells (**F**) and sorted B cells (**G**) shown using heatmaps. The color corresponds to the average gene expression levels in each cell type.

First, we successfully isolated splenic T and B cells from DHA-treated and control mice. scRNA-seq was then performed on these cells using the 10× Genomics Chromium system (Figure 2A). After removing cell doublets and filtering low-quality cells, we obtained 11,651 T cells and 13,481 B cells, providing a high-quality transcriptomic dataset. t-distributed stochastic neighbor embedding (t-SNE) clustering and differential gene expression analysis identified all clusters as T cells based on expression of markers Cd3d, Cd3e, and Cd3g (Figure 2B,D,F and Table S1), and B cells based on Cd79a, Cd79b, and Ms4a1 (Figure 2C,E,G and Table S2).

### 3.3. Re-Clustering of T Cells Illustrated Different Cell Proportions and Signaling Pathways in DHA-Treated and Control Mice

**Figure 3:**
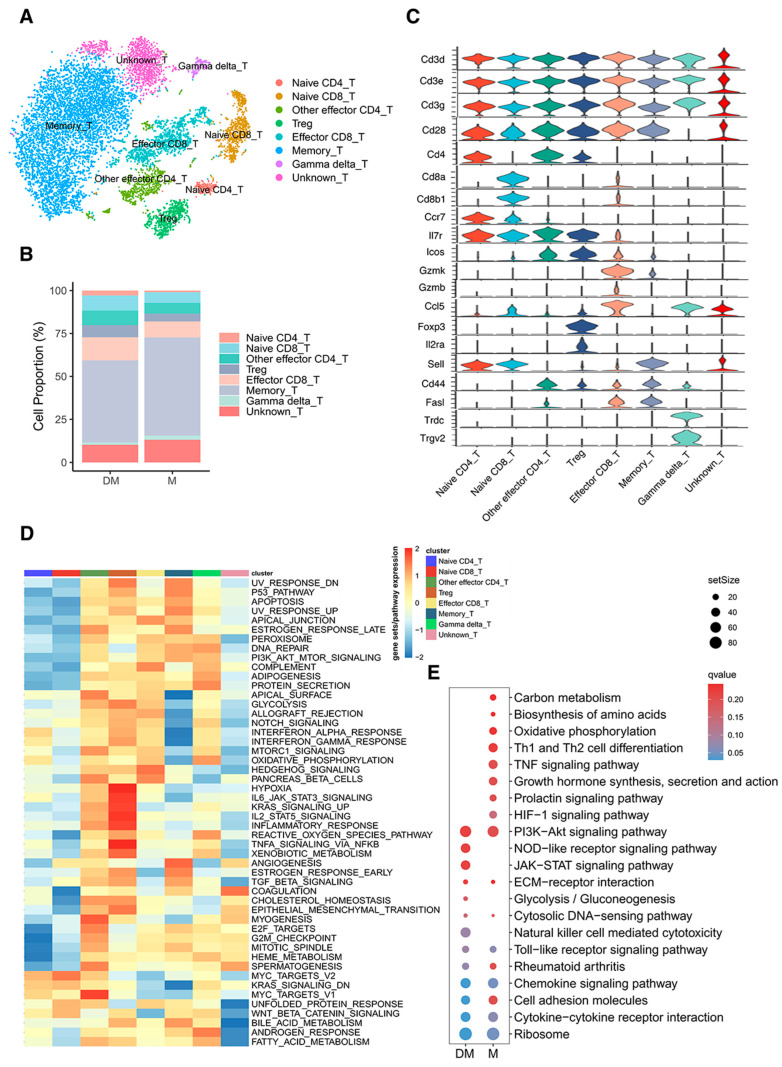
Comparison of T-cell subtypes from spleens of DHA-treated and control mice. (**A**): t-SNE plot showing T-cell subtypes from both DHA-treated and control mice. (**B**): Histogram depicting the relative proportions of T-cell subtypes in DHA-treated versus control mice. (**C**): Stacked violin plots illustrating the expression levels of selected genes used to define T-cell subtypes. (**D**): Heatmap of gene sets/pathways from GSEA based on hallmark gene sets for each T-cell subtype. (**E**): Bubble diagram highlighting KEGG pathways enriched in T cells from DHA-treated versus control mice. DM: DHA-treated mice; M: control mice.

After removing the batch effect, T-cell clusters were subdivided into eight subtypes (Figure 3A): naive CD4+ T cells with high expression of Cd4, Ccr7, and Sell; naive CD8+ T cells with high expression of Cd8a, Cd8b1, Ccr7, and Sell; effector CD4+ T cells including Treg cells with high expression of Cd4, Foxp3, Il2ra, and other effector CD4+ T cells with high expression of Cd4, Il7r, and Cd44; effector CD8+ T cells with high expression of Cd8a, Cd8b1, Gzmk, and Gzmb; memory T cells with high expression of Sell, Cd44, and Fasl; gamma delta T cells with high expression of Trdc and Trgv2; and unknown T cells with no specific gene expression except relatively high expression of Ccl5 (Figure 3C and Table S3).

We analyzed the proportions of different subtypes in DHA-treated and control mice (Figure 3B). In DHA-treated mice, the proportions of naive CD4+ and CD8+ T cells, Treg cells, and other effector CD4+ T cells were 2.9%, 8.8%, 7.1%, and 8.4%, respectively, compared to 1.0%, 6.3%, 6.2%, and 4.6% in control mice. Effector CD8+ T cells were 13.5% in DHA-treated mice versus 9.2% in controls. Conversely, memory T cells, gamma delta T cells, and unknown T cells were 47.7%, 1.4%, and 10.3% in DHA-treated mice, respectively, compared to 57.1%, 2.5%, and 13.2% in control mice. These results indicate a higher proportion of memory T cells and a lower proportion of naive and effector T cells in lupus mice, suggesting that naive and effector T cells might be exhausted, leading to a quicker immune response in lupus mice. DHA treatment appeared to mitigate this response to some extent.

To identify transcriptomic differences between T-cell subtypes, we conducted GSEA for hallmark gene sets based on DEGs. Notably, effector CD4+ and CD8+ T cells, memory T cells, and gamma delta T cells showed significant upregulation of signaling pathways compared to other subtypes, consistent with their more differentiated and active states (Figure 3D). GSEA for KEGG pathway analysis revealed significant upregulation of signaling pathways, including HIF-1 signaling, TNF signaling, and Th1 and Th2 cell differentiation in T cells of control mice (Figure 3E and Table S4), which are known to play important roles in immune response and inflammation [19,20].

### 3.4. Re-Clustering of B Cells Revealed Significant Reduction of Plasma Cells in Spleen of DHA-Treated Mice

**Figure 4:**
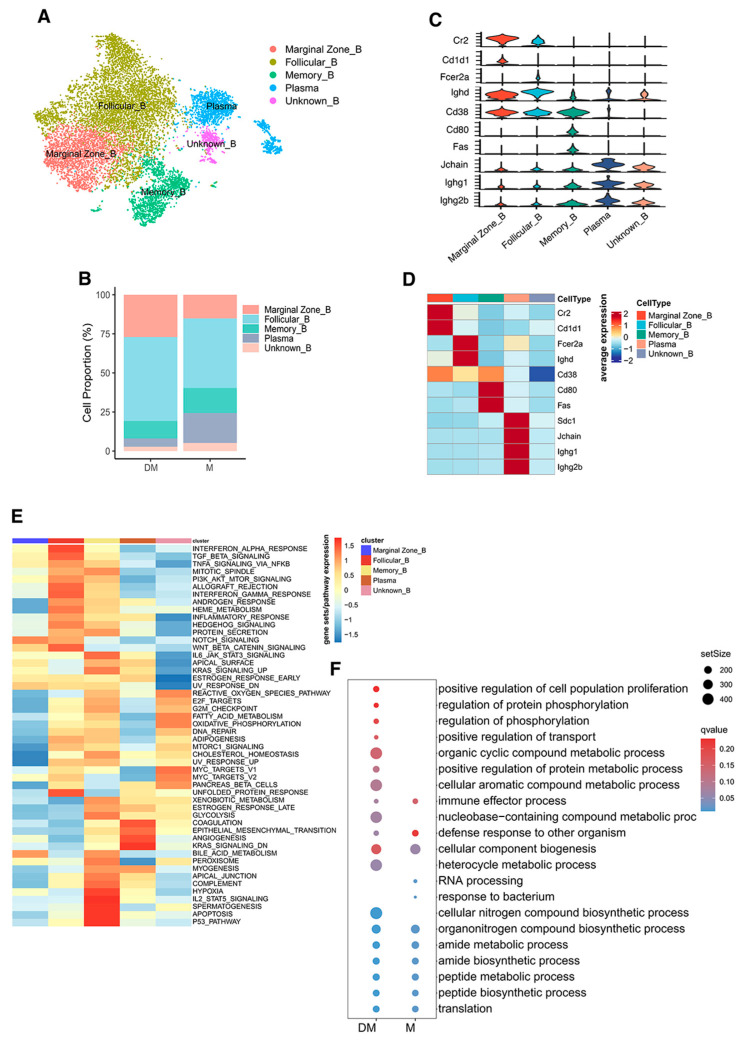
Comparison of B-cell subtypes in the spleen of DHA-treated and control mice. (**A**): t-SNE plot representing B-cell subtypes from DHA-treated and control mice. (**B**): Histogram showing the relative proportions of B-cell subtypes in DHA-treated versus control mice. (**C**,**D**): Stacked violin plots (**C**) and heatmap (**D**) displaying the expression levels of selected genes defining B-cell subtypes. (**E**): Heatmap of gene sets/pathways using GSEA based on hallmark gene sets for each B-cell subtype, performed using all differentially expressed genes. (**F**): Bubble diagram showing GO analysis enriched in B cells between DHA-treated and control mice. DM: DHA-treated mice; M: control mice.

B cells, the primary immune cells producing autoantibodies, play a crucial role in the pathogenesis of lupus. After removing the batch effect, we generated and labeled five subtypes for further analysis (Figure 4A): marginal zone B cells, characterized by high expression of Cr2 and Cd1d1; follicular B cells, characterized by high expression of Fcer2a and Ighd; memory B cells, characterized by high expression of Cd80 and Fas; plasma cells, characterized by high expression of Sdc1, Jchain, and immunoglobulin proteins such as Ighg1 and Ighg2b; and unknown B cells, an isolated cluster similar to plasma cells but with lower expression of Jchain, Ighg1, and Ighg2b (Figure 4C,D and Table S5). This suggests that unknown B cells might represent a population of short-lived plasma cells undergoing programmed cell death.

In DHA-treated mice, the proportions of marginal zone B cells, follicular B cells, memory B cells, plasma cells, and unknown B cells were 27.1%, 53.7%, 11.2%, 5.3%, and 2.7%, respectively, while in control mice, these proportions were 15.1%, 44.6%, 16.0%, 19.1%, and 5.2%, respectively (Figure 4B). The increased proportions of plasma cells, unknown B cells, and memory B cells in control mice indicate a more pronounced and rapid pathogenic immune response compared to DHA-treated mice.

GSEA analysis of hallmark gene sets showed significant upregulation of signaling pathways in follicular and memory B cells (Figure 4E). However, GSEA for KEGG pathway analysis identified only a limited number of significant signaling pathways (Figure S1 and Table S6). Additionally, further GSEA for GO analysis revealed distinct enrichment patterns between the two groups (Figure 4F and Table S7).

### 3.5. Pseudotime Trajectory Analysis Unveiled the Developmental and Molecular Characteristics of T-Cell and B-Cell Subtypes in Spleen

Next, we conducted pseudotime trajectory analysis on T-cell and B-cell subtypes to explore the developmental positions of unknown T and B cells and to identify key genes associated with specific cell differentiation using Monocle 2.

**Figure 5:**
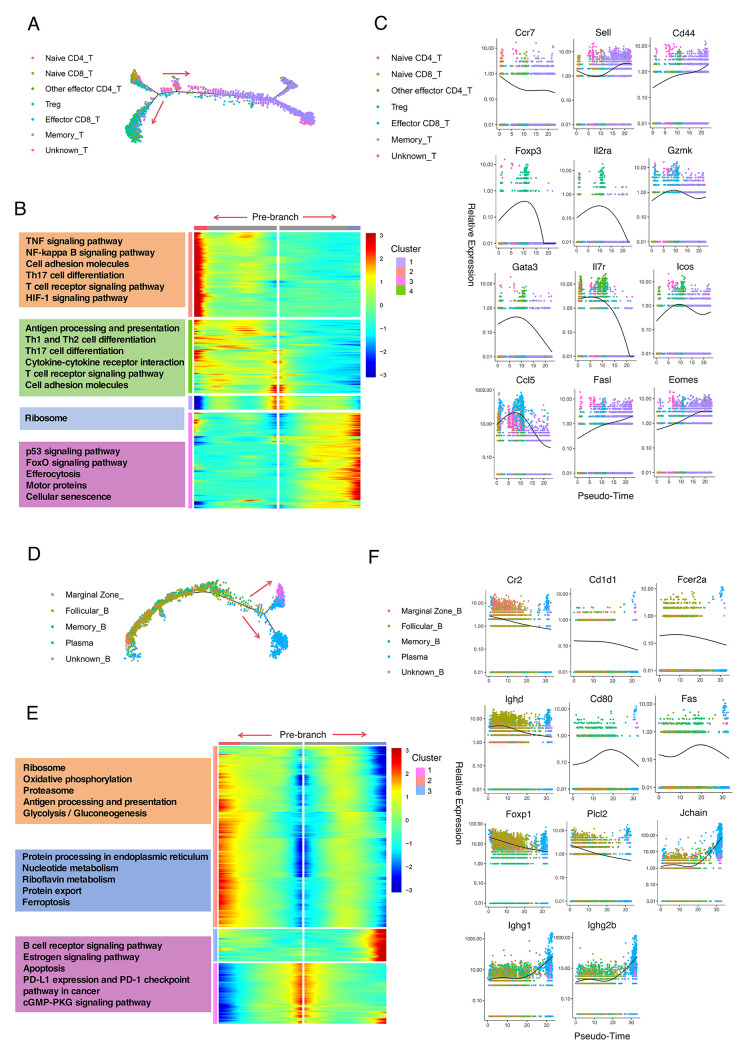
Trajectory analysis of T cells and B cells from the spleen. (**A**,**D**): Trajectory plots show pseudotime trajectory analysis of T-cell (**A**) and B-cell (**D**) subtypes using Monocle 2. Arrows indicate the possible differentiation direction. (**B**,**E**): Pseudotime expression modules of T cells (**B**) and B cells (**E**) were identified by Monocle 2, illustrating cell development at main branch points. Differences in KEGG enrichment pathways across different expression modules are shown on the left. (**C**,**F**): The expression values of representative marker genes and other specific genes are plotted along the pseudotime trajectory for T cells (**C**) and B cells (**F**).

For T cells, as shown in Figure 5A, all T-cell subtypes were organized into four main branches. Naive T cells (CD4+ and CD8+) predominantly occupied the early stages of development, while most effector T cells (CD4+ and CD8+) were situated at the terminal stages of the trajectory. Memory T cells were primarily located at the two other terminal stages. The analysis also revealed that the cluster of unknown T cells was mainly in an intermediate state between naive T cells and memory T cells, suggesting that these unknown T cells might be activated T cells undergoing programmed cell death. To further elucidate the biological processes involved in cell development at the main branch points, KEGG analysis was performed on four distinct gene expression modules (Figure 5B). Module 1 was highly enriched in unknown T cells. The gene sets in this module were predominantly involved in ribosomal processes, with several genes related to cell proliferation and apoptosis (e.g., Eef1g, Tpt1). These results support the hypothesis that the unknown T-cell cluster might represent apoptotic cells following activation. Module 2 contained genes mainly expressed in effector T cells. These genes were associated with pathways including TNF signaling, NF-kappa B signaling, cell adhesion molecules, Th17 cell differentiation, T-cell receptor signaling, and HIF-1 signaling. Module 3 included genes expressed in memory T cells, primarily encoding inhibitory signaling pathways such as p53 signaling, FoxO signaling, and efferocytosis. Module 4 comprised genes upregulated in pre-branch naive T cells and some effector T cells. This module was involved in antigen processing and presentation, Th1 and Th2 cell differentiation, Th17 cell differentiation, cytokine–cytokine receptor interactions, T-cell receptor signaling, and cell adhesion molecules. The expression values of representative marker genes and other specific genes in each cell type were further plotted along the pseudotime trajectory of T cells (Figure 5C). Collectively, these genes and signaling pathways might serve as critical regulators and targets in T-cell differentiation and the development of lupus.

For B cells, the pseudotime analysis revealed three main branches. Most marginal zone B cells and follicular B cells were located at the initial stage. Part of the marginal zone B cells and follicular B cells, along with the majority of memory B cells, were positioned at the intermediate stage. Plasma cells were observed at both terminal stages, which might represent short-lived and long-lived plasma cells, respectively. The unknown B cells were found at one of the terminal stages alongside plasma cells (Figure 5D). KEGG pathway enrichment was performed on three distinct gene expression modules to investigate the biological processes of B-cell development. Module 1 primarily included gene sets highly enriched in pre-branch marginal zone B cells, follicular B cells, and some memory B cells, which were predominantly in a mature and quiescent state. These genes encoded pathways related to B-cell receptor signaling, estrogen signaling, apoptosis, PD-L1 expression and PD-1 checkpoint pathway in cancer, protein processing in the endoplasmic reticulum, and cGMP-PKG signaling. Module 2 comprised genes mainly expressed in one terminal stage of plasma cells, encoding components related to the ribosome, oxidative phosphorylation, proteasome, and antigen processing and presentation. Module 3 included various immunoglobulin-coding genes and was associated with the other terminal stage of plasma cells, including the cluster of unknown B cells. This module was involved in protein folding, sorting and degradation, metabolism, and cell growth and death (Figure 5E). The expression patterns of marker genes and other specific genes for B-cell subtypes were further analyzed and are shown in Figure 5F.

### 3.6. DHA Might Inhibit B-Cell Differentiation into Plasma Cells by Regulating the Function of Effector CD4+T Cells in Lupus Spleen

**Figure 6:**
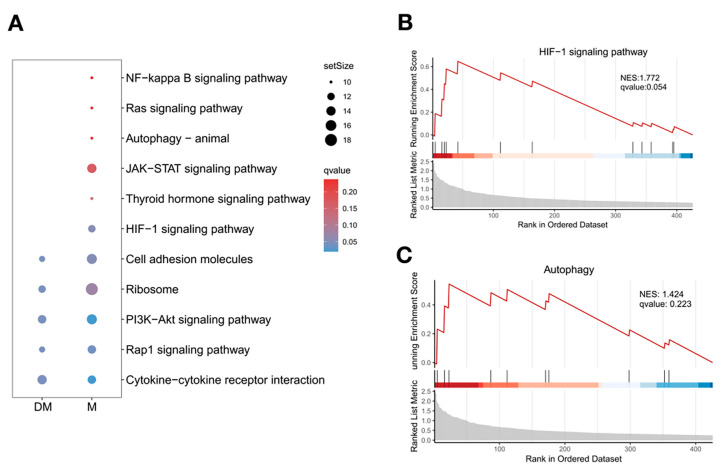
Comparison of signaling pathways in effector CD4+ T cells between DHA-treated and control mice. (**A**): A bubble diagram represents the KEGG pathways in effector CD4+ T cells from DHA-treated and control mice using the GSEA method. (**B**,**C**): Enrichment plots of HIF-1 signaling (**B**) and autophagy (**C**) in control mice. NES: normalized enrichment scores. DM: DHA-treated mice; M: control mice.

The results indicate a significant decrease in plasma cells in the spleen of DHA-treated mice. Effector CD4+ T cells were identified as crucial regulators of B-cell differentiation and activation. Therefore, we examined the KEGG signaling pathways in effector CD4+ T cells from DHA-treated mice and control mice using the GSEA method. Interestingly, several signaling pathways, including HIF-1 signaling and autophagy, were upregulated in effector CD4+ T cells from control mice compared to the DHA-treated group (Figure 6A). We further analyzed these two pathways using the normalized enrichment score (NES) (Figure 6B,C). The core enrichment for the HIF-1 signaling pathway included Igf1r, Bcl2, Prkca, HIF1α, and Il6ra, while the autophagy pathway included Igf1r, Bcl2, and HIF1α. HIF1α has been reported to be upregulated in the kidneys of LN patients and in peripheral CD4+ T cells of lupus patients [21,22]; however, the mechanisms behind the abnormal levels of HIF1α are still unclear. Our findings suggest that DHA might inhibit the function of effector CD4+ T cells by modulating the HIF-1 signaling pathway, specifically targeting HIF1α expression. Further studies are needed to verify this mechanism.

### 3.7. Different Interactions Were Found Between Effector CD4+T Cells and B Cells in DHA-Treated and Control Mice

**Figure 7:**
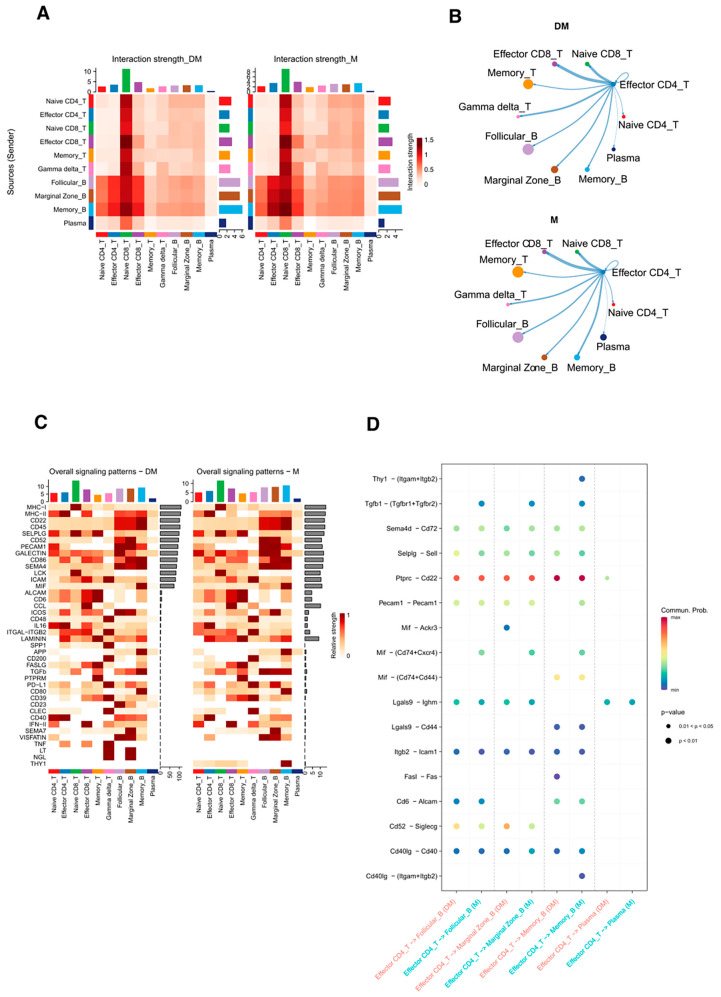
Ligand–receptor interactions between different cell types in DHA-treated and control mice. (**A**): Heatmap of interactions between cell types in DHA-treated and control mice. Column annotations represent cells from both groups. Colors indicate interaction scores. (**B**): Network diagram showing ligand–receptor interactions between effector CD4+ T cells and other cell types in DHA-treated and control mice. Connected lines represent communication between cells, with line thickness proportional to the number of ligand–receptor interaction events. (**C**): Signaling (outgoing and incoming) showing ligand–receptor interactions between T cells and B cells in DHA-treated and control mice. (**D**): Bubble charts representing interactions between effector CD4+ T cells and B-cell subtypes, with at least one set of cell pairs having a *p*-value less than 0.05. DM: DHA-treated mice; M: control mice.

Finally, we analyzed the expression levels of ligand–receptor interaction pairs between T cells and B cells in both groups using CellChat. Among all cell types, effector CD4+ T cells exhibited relatively higher interaction with memory B cells in control mice compared to the DHA-treated group (Figure 7A,B and Table S8). These findings support the notion that effector CD4+ T cells regulate B-cell function, while DHA appears to reduce the interaction between these two cell types.

We compared signaling changes (both outgoing and incoming) in T-cell and B-cell subtypes between the two groups and observed notable differences in signal intensity, particularly through the THY1, MIF, TGFβ, and PECAM1 pathways between effector CD4+ T cells and memory B cells (Figure 7C and Table S9). To further understand these interactions, we explored the receptor–ligand pairs mediating the communication. Our findings revealed that the ligands Thy1, Tgfβ1, Mif, and Pecam1 on effector CD4+ T cells interacted with memory B cells via receptors Itgam + Itgb2, Tgfbr1 + Tgfbr2, Cd74 + Cxcr4, and Pecam1 , respectively (Figure 7D).

## 4. Discussion

The mechanism by which DHA alleviates lupus symptoms remains incompletely understood. In our study, we utilized single-cell RNA sequencing (scRNA-seq) to compare the composition and interactions of splenic T cells and B cells in DHA-treated mice and untreated lupus mice. This approach provided a comprehensive understanding of cellular status, in vivo developmental trajectories, functional changes, and signaling pathways in different cell populations under DHA treatment.

We initially evaluated the therapeutic effects of DHA in MRL/lpr mice, a model of systemic lupus erythematosus (SLE). DHA treatment resulted in a reduction in hair loss and spleen size, as well as decreased kidney inflammation, consistent with the existing literature. However, no significant changes were observed in skin tissues, despite the reduction in hair loss in the DHA-treated group. This lack of significant change in skin tissues may be attributed to the biopsy sites being selected in regions with more severe inflammation in both groups. Previous studies have also reported reductions in serum levels of ANA, anti-ds-DNA, and anti-RNP/sm antibodies in lupus mice following DHA treatment [12,23].

Splenic T cells exhibit diverse subtypes with specific signaling pathways and functions. Our results indicate that DHA treatment decreased the proportion of memory T cells and unknown T cells. Memory T cells, which are known to increase in lupus mice, can respond more quickly and efficiently to their specific antigens, including auto-antigens [24]. The unknown T cells identified here may represent activated T cells undergoing programmed cell death after B-cell activation. These findings suggest that lupus mice have more active and rapid immune responses, while DHA treatment may partially reduce immune activation. Additionally, DHA treatment downregulated several inflammation-related pathways in T cells, further supporting its immunomodulatory effects. Notably, we observed that effector CD4+ T cells, crucial for regulating B-cell activation and autoantibody production, exhibited downregulated signaling pathways, including HIF-1 signaling and autophagy, in DHA-treated mice. Further characterization of these signaling pathways revealed three common core enrichment genes, including Hif1a (HIF1α). Hif1a (HIF1α) is a primary metabolic sensor involved in metabolic pathways that regulate immune cell function and inflammation [25,26,27]. Previous studies have linked Hif1a (HIF1α) to SLE, with higher levels observed in the urine of LN patients and peripheral blood CD4+ T cells of SLE patients [22,28]. Increased HIF-1α expression was noted in both glomerular and tubulointerstitial areas in LN patients and MRL/lpr lupus mice [21]. Silencing HIF-1α using RNAi techniques reduced serum levels of IL-17, proteinuria, and IgG and C3 deposits in the kidneys of MRL/lpr mice [29]. Our findings suggest that DHA significantly decreased HIF-1α expression in effector CD4+ T cells from the spleen of MRL/lpr mice. We speculate that DHA might inhibit CD4+ T-cell function by downregulating HIF-1 signaling pathways through HIF1a (HIF1α), potentially contributing to its therapeutic effects in lupus. A related study has shown that dihydroartemisinin suppresses glycolysis in LNCaP cells by inhibiting the PI3K/AKT pathway and downregulating HIF-1α expression in prostate cancer cells [30]. However, further studies are needed to fully elucidate this mechanism.

Moreover, our results demonstrated that DHA treatment reduced the proportions of plasma and memory B cells in lupus mice, indicating attenuation of the excessively pathogenic immune response. Additionally, the unknown B cells, potentially representing short-lived plasma cells undergoing programmed cell death, were also decreased in DHA-treated mice. We observed several changes in the biological processes of B cells in lupus mice following DHA treatment, which warrant further detailed investigation. Combining these findings with those from T cells, we hypothesize that DHA may inhibit B-cell differentiation into plasma cells by modulating effector CD4+ T cells.

Cell communication analysis revealed altered interactions between effector CD4+ T cells and B cells in lupus mice compared to controls, with DHA treatment reducing these interactions. Specifically, ligand–receptor pairs such as Thy1-(Itgam+Itgb2), Mif-(Cd74+Cxcr4), Tgfb1-(Tgfbr1+Tgfbr2), and Pecam1-Pecam1 showed higher mean expression levels in lupus mice than in DHA-treated mice. This suggests a potential mechanism by which DHA regulates T/B cell activation.

Our study has several limitations. We focused exclusively on splenic T and B cells, which did not provide information on interactions with other cell types such as dendritic cells or macrophages. Additionally, we used only one lupus mouse model and did not include TCR and BCR diversity sequencing. Furthermore, the lack of experimental validation for our findings is another limitation. Future studies should explore interactions with other cell types and confirm our results in other mouse models and lupus patients, if possible.

## 5. Conclusions

In conclusion, our findings reveal changes in cell proportions and signaling pathways in splenic T and B cells of lupus mice following DHA treatment. DHA may inhibit B-cell differentiation into plasma cells by regulating effector CD4+ T cells, potentially through influencing HIF1α and ligand–receptor pairs. These insights help us to gain a preliminary understanding of the mechanisms underlying DHA treatment in SLE. While the mechanism of DHA has been partially demonstrated in lupus mouse models, further verification is needed in other mouse models and human SLE.

## Data Availability

The raw data supporting the findings of this study are available in the public database under the BioProject accession number PRJNA1287573.

## Supplementary Materials

The following supporting information can be downloaded at https://www.mdpi.com/article/10.3390/cimb47070528/s1.
