# Supplementary material for: Dihydroartemisinin Alleviates the Symptoms of a Mouse Model of Systemic Lupus Erythematosus Through Regulating Splenic T/B-Cell Heterogeneity
Source: Curr Issues Mol Biol. 2025 Jul 9;47(7):528. doi: 10.3390/cimb47070528 (PMC12293267; doi:10.3390/cimb47070528)
Supplement: Supplementary file 1 [file cimb-47-00528-s001.zip › supplementary tables and figures/Figure legend-Fig. S1.pdf]

Suppl. Fig.S1

KEGG pathways in B cells between DHA-treated and control mice. Bubble diagram showing KEGG pathways enriched in B cells between DHA-treated and control mice. DM: DHA-treated mice; M: control mice
