# Supplementary material for: Dihydroartemisinin Alleviates the Symptoms of a Mouse Model of Systemic Lupus Erythematosus Through Regulating Splenic T/B-Cell Heterogeneity
Source: Curr Issues Mol Biol. 2025 Jul 9;47(7):528. doi: 10.3390/cimb47070528 (PMC12293267; doi:10.3390/cimb47070528)
Supplement: Supplementary file 1 [file cimb-47-00528-s001.zip › supplementary tables and figures/Table S1.pdf]

Suppl. Table S1 Maker genes in isolated T cells from spleen in both DHA-treated and control mice

gene	p_val	avg_log2FC	pct.1	pct.2	p_val_adj	cluster
Cd3e	1.00E-27	0.3571086	0.971	0.852	3.24E-23	5
Cd3e	1.43E-147	0.860103441	0.978	0.852	4.63E-143	6
Cd3e	6.98E-40	0.70986694	0.941	0.857	2.25E-35	7
Cd3e	1.45E-08	0.271191538	0.963	0.857	0.000467314	10
Cd3d	5.21E-28	0.359166183	0.954	0.831	1.68E-23	5
Cd3d	4.12E-73	0.642615116	0.952	0.831	1.33E-68	6
Cd3d	2.86E-09	0.316623886	0.93	0.836	9.22E-05	10
Cd3g	1.31E-44	0.286969688	0.925	0.809	4.21E-40	3
Cd3g	3.17E-51	0.505330586	0.947	0.812	1.02E-46	5
Cd3g	1.01E-216	1.419467267	0.977	0.811	3.25E-212	6
Cd3g	1.51E-63	1.205059541	0.97	0.817	4.87E-59	7
Cd3g	2.59E-18	0.435451369	0.979	0.817	8.38E-14	10
Cd19	0	1.55125435	0.797	0.001	0	12
Cd79a	0	4.139631751	0.949	0.016	0	12
Cd79a	0	1.844341547	0.8	0.019	0	14
Cd79a	1.33E-52	1.844197567	0.339	0.024	4.30E-48	15
Cd79b	0	3.407702425	0.975	0.059	0	12
Cd79b	3.85E-107	1.071706422	0.62	0.063	1.24E-102	14
Ms4a1	0	3.237299511	0.907	0.003	0	12
Fcgr3	0	2.67711786	0.429	0.006	0	8
Fcgr3	4.04E-266	2.321768183	0.909	0.013	1.30E-261	16
Cd14	4.29E-44	2.904578619	0.636	0.043	1.38E-39	16
Fcer1g	0	4.253178777	0.735	0.015	0	8
Fcer1g	9.22E-204	2.852394794	0.695	0.026	2.98E-199	15
Fcer1g	1.93E-165	4.162927572	1	0.027	6.23E-161	16
