# Supplementary material for: Dihydroartemisinin Alleviates the Symptoms of a Mouse Model of Systemic Lupus Erythematosus Through Regulating Splenic T/B-Cell Heterogeneity
Source: Curr Issues Mol Biol. 2025 Jul 9;47(7):528. doi: 10.3390/cimb47070528 (PMC12293267; doi:10.3390/cimb47070528)
Supplement: Supplementary file 1 [file cimb-47-00528-s001.zip › supplementary tables and figures/Table S2.pdf]

Suppl. Table S2 Maker genes in isolated B cells from spleen in both DHA-treated and control mice

gene	p_val	avg_log2FC	pct.1	pct.2	p_val_adj	cluster
Cd3d	0	3.119489598	0.911	0.006	0	10
Cd3e	0	3.004072554	0.889	0.002	0	10
Cd3g	0	2.903455992	0.889	0.026	0	10
Cd79b	3.66E-89	0.257900886	0.994	0.856	1.18E-84	0
Cd79b	1.18E-305	0.872640027	1	0.883	3.81E-301	3
Ms4a1	3.69E-138	0.353033248	0.998	0.8	1.19E-133	1
Ms4a1	4.74E-196	0.80504931	0.995	0.832	1.53E-191	3
Cr2	0	1.737899335	0.945	0.435	0	1
Fcgr3	0	2.523350029	0.336	0.007	0	7
Fcgr3	7.93E-196	1.544782831	0.531	0.017	2.56E-191	11
Fcer1g	0	4.096790656	0.699	0.06	0	7
Fcer1g	1.13E-85	2.766105852	0.734	0.08	3.63E-81	11
