# Supplementary material for: Dihydroartemisinin Alleviates the Symptoms of a Mouse Model of Systemic Lupus Erythematosus Through Regulating Splenic T/B-Cell Heterogeneity
Source: Curr Issues Mol Biol. 2025 Jul 9;47(7):528. doi: 10.3390/cimb47070528 (PMC12293267; doi:10.3390/cimb47070528)
Supplement: Supplementary file 1 [file cimb-47-00528-s001.zip › supplementary tables and figures/Table S3.pdf]

Suppl. Table S3 Maker genes in T cell subtypes from spleen in both DHA-treated and control mice

gene	p_val	avg_log2FC	pct.1	pct.2	p_val_adj	cluster
Cd3d	1.11E-22	0.311561569	0.957	0.863	3.60E-18	Treg
Cd3d	2.36E-86	0.476987489	0.956	0.858	7.61E-82	Effector CD8_T
Cd3e	5.92E-22	0.307690576	0.974	0.886	1.91E-17	Treg
Cd3e	3.45E-127	0.574888713	0.974	0.881	1.11E-122	Effector CD8_T
Cd3e	5.92E-41	0.705621717	0.977	0.89	1.91E-36	Gamma delta_T
Cd3g	2.90E-46	0.468518817	0.949	0.844	9.38E-42	Treg
Cd3g	2.10E-59	1.174886251	0.991	0.848	6.78E-55	Gamma delta_T
Cd3g	1.04E-208	0.998194842	0.972	0.835	3.37E-204	Effector CD8_T
Cd4	1.65E-57	0.665928795	0.665	0.193	5.34E-53	Naive CD4_T
Cd4	0	1.473605581	0.759	0.159	0	Other effector CD4_T
Cd4	6.20E-110	0.502156587	0.573	0.18	2.00E-105	Treg
Cd8a	0	1.906474886	0.84	0.054	0	Naive CD8_T
Cd8a	7.88E-83	0.695234393	0.273	0.093	2.54E-78	Effector CD8_T
Cd8b1	0	1.945772643	0.869	0.102	0	Naive CD8_T
Cd8b1	8.53E-54	0.756446727	0.299	0.142	2.75E-49	Effector CD8_T
Ccr7	8.90E-98	1.167581671	0.749	0.197	2.87E-93	Naive CD4_T
Ccr7	4.13E-188	0.703255406	0.616	0.174	1.33E-183	Naive CD8_T
Il7r	1.40E-63	0.990933363	0.767	0.24	4.52E-59	Naive CD4_T
Il7r	0	1.087442539	0.824	0.203	0	Naive CD8_T
Il7r	1.85E-295	2.119781134	0.734	0.211	5.97E-291	Other effector CD4_T
Il7r	9.92E-276	1.473336356	0.836	0.213	3.20E-271	Treg
Icos	1.06E-91	0.931694639	0.656	0.34	3.41E-87	Other effector CD4_T
Icos	5.76E-128	1.239878159	0.721	0.341	1.86E-123	Treg
Icos	6.36E-20	0.276740744	0.47	0.349	2.05E-15	Effector CD8_T
Gzmk	0	1.613155246	0.809	0.295	0	Effector CD8_T
Gzmb	3.90E-123	1.168695552	0.332	0.104	1.26E-118	Effector CD8_T
Ccl5	1.18E-88	1.483506597	0.944	0.403	3.80E-84	Gamma delta_T
Ccl5	0	4.359388155	0.851	0.357	0	Effector CD8_T
Foxp3	0	2.2429807	0.761	0.008	0	Treg
Il2ra	0	1.788056505	0.504	0.036	0	Treg
Sell	0	1.084531711	0.865	0.369	0	Memory_T
Cd44	1.27E-295	0.596543266	0.692	0.348	4.11E-291	Memory_T
Fasl	0	0.861680125	0.736	0.28	0	Memory_T
Trdc	0	4.03154149	0.958	0.024	0	Gamma delta_T
Trgv2	0	2.005294928	0.704	0.02	0	Gamma delta_T
