# Supplementary material for: Dihydroartemisinin Alleviates the Symptoms of a Mouse Model of Systemic Lupus Erythematosus Through Regulating Splenic T/B-Cell Heterogeneity
Source: Curr Issues Mol Biol. 2025 Jul 9;47(7):528. doi: 10.3390/cimb47070528 (PMC12293267; doi:10.3390/cimb47070528)
Supplement: Supplementary file 1 [file cimb-47-00528-s001.zip › supplementary tables and figures/Table S4.pdf]

Suppl. Table S4 KEGG pathways enriched in T cells from DHA-treated versus control mice

ID	Group	Description	pvalue	core_enrichment
mmu03010	DM	Ribosome	0.000999001	Rps19/Rpl12/Rps20/Rps4x/Rpl36a/Rpl14/Rps11/Rps6/Rps2/ Rpl3/Rpl26/Rpl10/Rpl5/Rps9/Rpl8/Rps24/Rpsa/Rps21/Rps29/ Rps7/Rpl35/Rps8/Rps16/Rpl17/Rps18/Rpl7/Rps28/Rpl23/ Rpl36/Rps23/Rpl38/Rpl9/Rpl6/Rps13/Rps26/Rpl10a/Uba52/ Rpl24/Rpl32/Rpl13/Rps5/Rps27a/Rpl18/Rps27/Rpl18a/Rplp1/ Rpl39/Rpl15/Rpl30/Rpl19/Rpl37a/Rpl7a/Rpl11/Rps25/Rps10/ Rpl37/Rpl35a/Rps3a1/Rpl27a/Rplp2/Rps3/Rpl23a/Rps15a/ Rpl21/Rpl31/Rps14/Rpl13a/Rpl22/Fau/Rps15/Rpl28/Rplp0/ Rps12/Rpl27/Rpl34
mmu04060	DM	Cytokine-cytokine receptor interaction	0.001	Ccl5/Ccl4/Xcl1/Ccl3/Tnfsf8/Ccr5/Il2ra/Tnfsf10/Tnfrsf18/Ccr2/ Ccr7/Ifng/Tnfrsf1b/Tnfrsf9/Il6ra/Il18r1/Il7r/Fasl/Tnfsf11/Ifngr1/ Il2rb/Acvr2b/Ltb/Il10ra/Cxcr5/Tgfbr1/Cd40lg/Ifngr2/Ifnar2/Il10rb
mmu04514	DM	Cell adhesion molecules	0.001008065	Itgb1/Ptprm/Cd8b1/Cd8a/Alcam/Ctla4/Itgb8/Sell/Pecam1/Itgb2 /H2-Aa/Itgav/Pdcd1lg2/Itga6/Icos/H2-Eb1/Cd40lg/Pdcd1/Itgal/ Cd226/Cd4/Sdc4/Sdc1/H2-T23/H2-K1/Itgb7/Cd28/Cd2/Spn
mmu04062	DM	Chemokine signaling pathway	0.001	Ccl5/Ccl4/Xcl1/Ccl3/Plcg2/Ccr5/Stat1/Hck/Ccr2/Plcb4/Ccr7/ Gnaq/Foxo3/Tiam1/Lyn
mmu05323	DM	Rheumatoid arthritis	0.003102378	Ccl5/Ccl3/Ctla4/Jun/Ifng/Itgb2/Atp6v0a2/Fos/H2-Aa/Tnfsf11 /Ltb/H2-Eb1
mmu04620	DM	Toll-like receptor signaling pathway	0.004136505	Ccl5/Ccl4/Ccl3/Stat1/Jun/Fos/Spp1
mmu04650	DM	Natural killer cell mediated cytotoxicity	0.004004004	Cd244a/Klrc1/Plcg2/Tnfsf10/Klrc2/Lat2/Ifng/Prkca/Itgb2/Gzmb/ Klrd1/Fasl/Ifngr1/Prf1/Fyn/Ppp3ca/Cd48/Hcst/Sh2d1a/Casp3/ Ifngr2/Ptpn11/Ifnar2/Itgal/Sh3bp2/Nfatc1/Klrk1/Pik3cd
mmu04623	DM	Cytosolic DNA-sensing pathway	0.008820287	Ccl5/Ccl4/Samhd1
mmu00010	DM	Glycolysis / Gluconeogenesis	0.011363636	Ldhb/Tpi1/Gpi1/Pkm/Dld/Pgm2/Acss2/Pfkp/Aldoa/Pgam1/Acss1/Pdha1
mmu04512	DM	ECM-receptor interaction	0.019480519	Itgb1/Lamc1/Itgb8/Spp1/Itgav/Itga6/Cd44/Sdc4/Sdc1/Itgb7/Cd47
mmu04630	DM	JAK-STAT signaling pathway	0.022110553	Il2ra/Stat1/Socs3/Ifng/Stat4/Il6ra/Bcl2/Socs2/Il7r/Cish/Ifngr1/Il2rb/ Socs1/Il10ra/Akt3/Ifngr2/Ptpn11/Ifnar2/Il10rb/Stat5b
mmu04621	DM	NOD-like receptor signaling pathway	0.023069208	Ccl5/Stat1/Jun/Plcb4/Mcu/P2rx7/Nek7/Bcl2/Gbp7/Gbp2/Tnfaip3/ Birc2/Birc3/Irgm2
mmu04151	DM	PI3K-Akt signaling pathway	0.02	Itgb1/Il2ra/Myb/Lamc1/Igf1r/Itgb8/Pik3ap1/Il6ra/Prkca/Hsp90b1/ Bcl2/Rps6/Foxo3/Cdk6/Spp1/Il7r/Fasl/Sgk1/Itgav/Il2rb/Itga6/Akt3/ Pik3r5/Pten
mmu03010	M	Ribosome	0.000999001	Rps19/Rpl3/Rpl36a/Rps20/Rpl12/Rps2/Rpl14/Rpl10/Rpl35/Rps9/ Rps6/Rps11/Rps4x/Rpsa/Rps18/Rpl32/Rps24/Rps28/Rps8/Rpl5/ Rps26/Rps7/Rpl23/Rpl17/Rps5/Rpl10a/Rpl7a/Rps15a/Rpl39/

				Rps21/Rps29/Rpl6/Rpl8/Rps23/Rpl13/Rplp1/Rps13/Rpl26/Rpl36/ Rpl9/Rps25/Rps16/Rpl37/Rps12/Uba52/Rps14/Rpl24/Rpl23a/Rpl7/ Rplp0/Rpl27a/Rpl18a/Rpl28/Rpl30/Rpl22/Rps27a/Rpl15/ Rpl38/Rpl11/Rps10/Rps3a1/Rps3/Rpl18/Rpl19/Rps27/ Rpl35a/Rpl37a/Rpl13a/Rpl31/Rpl21/Rplp2/Rpl34/Fau
mmu04062	M	Chemokine signaling pathway	0.001001001	Ccl5/Xcl1/Ccl4/Ccl3/Plcg2/Ccr5/Hck/Gnaq/Tiam1/Plcb4/ Stat1/Ccr2/Cxcr6/Lyn/Ccr7/Pik3cd/Stat5b/Foxo3/Cxcr5
mmu04620	M	Toll-like receptor signaling pathway	0.002074689	Ccl5/Ccl4/Ccl3/Jun/Stat1/Fos
mmu04060	M	Cytokine-cytokine receptor interaction	0.002004008	Ccl5/Xcl1/Ccl4/Ccl3/Ccr5/Tnfrsf1b/Il6ra/Ifng/Tnfrsf9/Il2rb/ Ccr2/Il7r/Cxcr6/Ccr7/Tnfrsf4/Il18r1/Fasl/Tnfsf11/Cxcr5/ Cd40lg/Il2ra/Acvr2a
mmu04066	M	HIF-1 signaling pathway	0.005122951	Igf1r/Plcg2/Bcl2/Prkca/Hif1a/Il6ra/Ifng/Rps6
mmu04623	M	Cytosolic DNA-sensing pathway	0.009248555	Ccl5/Ccl4/Samhd1
mmu05323	M	Rheumatoid arthritis	0.015608741	Ccl5/Ccl3/Jun/Ifng/Fos/Itgb2
mmu04917	M	Prolactin signaling pathway	0.013374486	Socs3/Stat1/Fos/Socs1/Socs2/Cish/Pik3cd/Tnfsf11/ Stat5b/Foxo3
mmu04935	M	Growth hormone synthesis, secretion and action	0.026369168	Plcg2/Socs3/Prkca/Gnaq/Plcb4/Stat1/Fos/Socs1/Socs2/Junb/ Pik3cd/Stat5b/Itpr1
mmu04514	M	Cell adhesion molecules	0.023115578	Itgb1/Ptprm/Cd8a/Cd8b1/Itgb8/Alcam/Icos/Itgb2/Sell/Cd40lg/ Itga6/Cd2/Pdcd1lg2/Tigit/H2-Eb1/Neo1/Pecam1/Sdc4/H2-K1/ Cd4/Spn/Cd226/Icam1/Ctla4/Itgal/Vsir
mmu04668	M	TNF signaling pathway	0.026130653	Ccl5/Rps6ka5/Jun/Socs3/Tnfrsf1b/Fos/Ifi47/Il18r1/Junb/Pik3cd/ Birc3/Itch/Tnfaip3/Nfkbia/Birc2
mmu04151	M	PI3K-Akt signaling pathway	0.017982018	Itgb1/Igf1r/Itgb8/Lamc1/Bcl2/Prkca/Myb/Il6ra/Rps6/Il2rb/Il7r/ Cdk6/Sgk1/Hsp90b1/Fasl/Pik3cd/Ret/Foxo3/Il2ra/Itga6
mmu04658	M	Th1 and Th2 cell differentiation	0.034170854	Jun/Stat4/Ifng/Stat1/Runx3/Gata3/Il2rb/Fos/Maml2/Rbpj/Stat5b/ Il2ra/Ppp3cc/Nfkbia/H2-Eb1/Ifngr2/Maf/Ppp3ca/Il2rg/Ifngr1/ Cd3g/Cd4
mmu00190	M	Oxidative phosphorylation	0.04	Cox8a/Cox7a2l/Cox17/Cox10/Uqcrh/Cox7b/Atp6v0e/Ndufa6/ Atp6v1f/Ndufa2/Ndufa10/Cox5a/Cox6c/Cox4i1/Ndufb11/Ndufc2/ Uqcr10/Cox6b1/Atp6v1d/Atp6v0b/Cox7a2/Ndufa3/Ndufa13/ Uqcrb/Ndufa5/Atp6v1h/Ndufb5/Ndufa4
mmu04512	M	ECM-receptor interaction	0.04416761	Itgb1/Itgb8/Lamc1
mmu01230	M	Biosynthesis of amino acids	0.044642857	Got1/Ass1/Tpi1/Idh2/Pgam1/Pfkl/Pkm/Taldo1/Pgk1/Eno1/ Idh3a/Aldoa
mmu01200	M	Carbon	0.042553191	Glud1/Got1/Tpi1/Gpi1/Idh2/Pgam1/Me2/Acss2/Mdh1/Acss1/Pfkl/

metabolism	Pkm/Taldo1/Pgk1/Eno1/Idh3a/Idnk/Suclg2/Aldoa

DM: DHA-treated mice; M: control mice
