# Supplementary material for: Dihydroartemisinin Alleviates the Symptoms of a Mouse Model of Systemic Lupus Erythematosus Through Regulating Splenic T/B-Cell Heterogeneity
Source: Curr Issues Mol Biol. 2025 Jul 9;47(7):528. doi: 10.3390/cimb47070528 (PMC12293267; doi:10.3390/cimb47070528)
Supplement: Supplementary file 1 [file cimb-47-00528-s001.zip › supplementary tables and figures/Table S5.pdf]

Suppl. Table S5 Maker genes in B cell subtypes from spleen in both DHA-treated and control mice

gene	p_val	avg_log2FC	pct.1	pct.2	p_val_adj	cluster
Cr2	0	1.729918941	0.959	0.48	0	Marginal Zone_B
Cd1d1	7.28E-291	0.507396455	0.487	0.155	2.35E-286	Marginal Zone_B
Fcer2a	3.19E-283	0.611191342	0.297	0.06	1.03E-278	Follicular_B
Ighd	0	1.490894754	0.969	0.551	0	Follicular_B
Cd38	2.11E-91	0.318732774	0.943	0.715	6.80E-87	Marginal Zone_B
Cd38	7.59E-33	0.258303479	0.897	0.741	2.45E-28	Memory_B
Cd80	0	0.904753552	0.516	0.039	0	Memory_B
Fas	0	0.702742309	0.502	0.119	0	Memory_B
Jchain	0	5.245352839	0.846	0.577	0	Plasma
Ighg1	5.66E-284	4.488017373	0.764	0.509	1.83E-279	Plasma
Ighg2b	1.13E-211	4.545448385	0.706	0.56	3.63E-207	Plasma
