# Supplementary material for: Dihydroartemisinin Alleviates the Symptoms of a Mouse Model of Systemic Lupus Erythematosus Through Regulating Splenic T/B-Cell Heterogeneity
Source: Curr Issues Mol Biol. 2025 Jul 9;47(7):528. doi: 10.3390/cimb47070528 (PMC12293267; doi:10.3390/cimb47070528)
Supplement: Supplementary file 1 [file cimb-47-00528-s001.zip › supplementary tables and figures/Table S6.pdf]

Suppl. Table S6 KEGG pathways enriched in B cells from DHA-treated versus control mice

ID	Group	Description	pvalue	core_enrichment
mmu03010	DM	Ribosome	0.001	Rps26/Rpl41/Rpl3/Rpl35/Rps27a/Rpl22l1/Rps28/Rpl15/Rpl28/Rpl24/Rpl11/Uba52/Rps5/Rpl37/Rps17/Rpl18/Rpl36/Rpl6/Rps27l/Rpl27a/Rpl37a/Rpl36al/Rpl14/Rpl31/Rpl22/Rpl34/Rpl7a/Rps4x/Rpl7/Rps8/Rplp2/Rps23/Rps6/Rpl27/Rpl9/Rps3a1/Rps29/Rpl21/Rps25/Rpl19/Rpl38/Rpl29/Rps3/Rps13/Rpl30/Rps24/Rps10/Rps9/Rpl26/Rpl35a/Rps16/Rpl8/Rpl18a/Rps21/Rpl4/Rps14/Rpl5/Mrpl33/Rpl23a/Rpl10/Fau/Mrpl17/Mrps7/Rps15
mmu03010	M	Ribosome	0.000999001	Rps26/Rplp0/Rps12/Rpl10a/Rps2/Rpl35/Rpl41/Rpl3/Rpl22l1/Rpl36a/Rpl24/Rpl15/Rpl11/Rpl28/Rps27a/Rpl22/Uba52/Rpl39/Rps11/Rplp1/Rps5/Rps28/Rpl27a/Rpl27/Rpl7/Rpl32/Rps18/Rpl37/Rps4x/Rplp2/Rpl23a/Rpl7a/Rpl37a/Rpl6/Rpl18/Rps25/Rps17/Rpl36/Rpl26/Rpl21/Rps15a/Rps20/Rpl5/Rpl13/Rps8/Rpl23/Rps6/Rps19/Rps23/Rpl30/Rpsa/Rps3a1/Rps9/Rpl19/Rpl31/Rpl34/Rps16/Rps7/\Rps29/Rps13/Rpl14/Rpl35a/Rpl38/Rps21/Rps3/Rpl4/Rps24/Rps10/Rpl18a/Rpl17/Rpl9/Rpl8/Rpl29/Rpl10/Rps14/Rps15/Fau
mmu00190	M	Oxidative phosphorylation	0.001038422	Cox6c/Cox5a/Cox8a/Ndufa4/Uqcr10/Cox6b1/Cox7c/Cox7b/Cox17/Uqcrh/Cox5b/Cox4i1/Cox7a2/Ndufa7/Ndufa2/Ndufa6/Atp6v1f
mmu04714	M	Thermogenesis	0.001013171	Actg1/Cox6c/Cox5a/Cox8a/Ndufa4/Uqcr10/Actb/Cox6b1/Cox7c/Cox7b/Cox17/Rps6/Uqcrh/Cox5b/Cox4i1/Cox7a2/Ndufa7/Ndufa2/Ndufa6/Grb2
mmu03040	DM	Spliceosome	0.004127967	Hspa8/Snrpd1/Snrpf/Snrpg/Snrpe/Hnrnpa1/Alyref/Snrpd2/Snrpb/Snu13/Lsm3/Lsm2/Txnl4a/Hnrnpk/Srsf3/Snrpa1/Lsm4/Lsm5/Sf3b5/Magoh/Lsm6/Pcbp1/Snrpa
mmu00190	DM	Oxidative phosphorylation	0.003006012	Cox5a/Cox6c/Ndufa4/Uqcr10/Cox7a2/Cox5b/Cox4i1/Cox7b/Uqcrh/Cox7c/Cox6b1/Cycs/Uqcrq/Ndufa2/Uqcrb/Ndufa8/Ndufb5/Ndufb2/Ndufv3/Uqcr11/Ndufc1/Ndufs6/Ndufa7/Cox17/Ndufab1/Ndufb8/Ndufb7/Ndufb9/Ndufa12/Ndufs5/Ndufs8/Atp6v1f/Ndufa11
mmu04935	DM	Growth hormone synthesis, secretion and action	0.033707865	Itpr1/Akt3/Map2k6/Pik3cd/Cacna1s/Socs1/Gnas/Fos/Gnai2

DM: DHA-treated mice; M: control mice
