# Supplementary material for: Dihydroartemisinin Alleviates the Symptoms of a Mouse Model of Systemic Lupus Erythematosus Through Regulating Splenic T/B-Cell Heterogeneity
Source: Curr Issues Mol Biol. 2025 Jul 9;47(7):528. doi: 10.3390/cimb47070528 (PMC12293267; doi:10.3390/cimb47070528)
Supplement: Supplementary file 1 [file cimb-47-00528-s001.zip › supplementary tables and figures/Table S7.pdf]

Suppl. Table S7 GO analysis enriched in B cells from DHA-treated versus control mice

ID	Group	Description	pvalue	core_enrichment
GO:0006412	DM	translation	0.000999001	Rpl31/App/Eif4a1/Npm1/Rbm3/Rpl18/Rpl19/Rpl21/Rpl22/Rpl26/Rpl27/Rpl28/Rpl29/Rpl30/Rpl37a/Rpl6/Rpl7/Rpl9/Rps17/Rps24/Rps29/Rps3a1/Rps4x/Rps5/Rps6/Rps8/Uba52/Ybx1/Rpl27a/Rps3/Rpl7a/Rpl3/Rps26/Eif5a/Rps28/Rpl36/Rps23/Rpl15/Rpl35/Rpl11/Rps10/Rpl14/Rplp2/Rpl37/Rpl38/Rps27l/Rpl41/Rpl22l1/Rps13/Rpl24/Rpl34/Rps25/Rps9/Rps27a
GO:0043043	DM	peptide biosynthetic process	0.000999001	Rpl5/Rpl10/Rpl31/App/C1qbp/Cdk4/Eef2/Eif4a1/Fau/Rack1/Prmt1/Ncl/Npm1/Pa2g4/Ppp1ca/Rbm3/Rpl18/Rpl19/Rpl21/Rpl22/Rpl26/Rpl27/Rpl28/Rpl29/Rpl30/Rpl37a/Rpl6/Rpl7/R
GO:0006518	DM	peptide metabolic process	0.000999001	Rpl5/Rpl10/Rpl31/Apoe/App/C1qbp/Cdk4/Eef2/Eif4a1/Fau/Rack1/Prmt1/Ncl/Npm1/Pa2g4/Ppp1ca/Rbm3/Rpl18/Rpl19/Rpl21/Rpl22/Rpl26/Rpl27/Rpl28/Rpl29/Rpl30/Rpl37a/Rpl6/Rpl7/Rpl9/Rps14/Rps15/Rps16/Rps17/Rps24/Rps29/Rps3a1/Rps4x/Rps5/Rps6/Rps8/Sorl1/Uba52/Ybx1/Rpl27a/Rpl23a/Rpl8/Rps3/Rpl7a/Rpl3/Rps26/Mrpl17/Eif5a/Mrps7/Rps28/Rpl36/Eif3i/Rpl35a/Eif3f/Chchd1/Rps23/Rpl15/Rps21/Rpl35/Eef1d/Mrpl33/Serbp1/Rpl11/Rps10/Rpl14/Eef1g/Rplp2/Rpl37/Rpl38/Rpl4/Rps27l/Rpl41/Rpl22l1/Rps13/Rpl24/Rpl34/Mrpl52/Eif3k/Rps25/Rpl18a/Rps9/Rps27a/Immp2l
GO:0043604	DM	amide biosynthetic process	0.000999001	Rpl5/Rpl10/Rpl31/App/Ass1/C1qbp/Cdk4/Eef2/Eif4a1/Fau/Rack1/Prmt1/Ncl/Npm1/Pa2g4/Ppp1ca/Rbm3/Rpl18/Rpl19/Rpl21/Rpl22/Rpl26/Rpl27/Rpl28/Rpl29/Rpl30/Rpl37a/Rpl6/Rpl7/Rpl9/Rps14/Rps15/Rps16/Rps17/Rps24/Rps29/Rps3a1/Rps4x/Rps5/Rps6/Rps8/Uba52/Ybx1/Rpl27a/Rpl23a/Rpl8/Rps3/Rpl7a/Rpl3/Rps26/Mrpl17/Eif5a/Mrps7/Rps28/Rpl36/Eif3i/Rpl35a/Eif3f/Chchd1/Rps23/Rpl15/Rps21/Rpl35/Eef1d/Mrpl33/Serbp1/Rpl1
GO:0043603	DM	amide metabolic process	0.000999001	Rpl5/Rpl10/Rpl31/Apoe/App/Ass1/C1qbp/Cdk4/Eef2/Eif4a1/Fau/Rack1/Prmt1/Ncl/Npm1/Pa2g4/Ppp1ca/Rbm3/Rpl18/Rpl19/Rpl21/Rpl22/Rpl26/Rpl27/Rpl28/Rpl29/Rpl30/Rpl37a/Rpl6/Rpl7/Rpl9/Rps14/Rps16/Rps17/Rps24/Rps29/Rps3a1/Rps4x/Rps5/Rps6/Rps8/St6gal1/Sorl1/Uba52/Ybx1/Rpl27a/Rpl23a/Rpl8/Rps3/Rpl7a/

GO:1901566	DM	organonitrogen compound biosynthetic process	0.000999001	Adk/Aldoa/Apoe/App/Aprt/Ass1/Bzw2/C1qbp/Ccnd3/Cdk4/Cers6/Chchd1/Derl3/Dut/Eef1d/Eef1g/Eef2/Eif1/Eif1ax/Eif2s2/Eif3f/Eif3h/Eif3i/Eif3k/Eif3l/Eif4a1/Eif5a/Eno1/Fau/Fh1/Hif1a/Impdh2/Magoh/Mgat5/Mrpl12
GO:0044271	DM	cellular nitrogen compound biosynthetic process	0.000999001	Rpl5/Eaf2/Sri/Dut/Nr3c2/Rpl10/Rpl31/Adk/Aldoa/Apex1/Birc5/Apoe/App/Aprt/Ass1/Bach2/Phb2/Bmp6/Bmpr1a/C1qbp/Anxa2/Cbfa2t3/Ccnd3/Cct2/Cct3/Cct4/Cct5/Cct6a/Cct7/C
GO:0006412	M	translation	0.000999001	Rpl5/Rpl10/Rpl31/App/Rplp0/C1qbp/Eef1a1/Eif4a1/Fau/Rack1/Rpsa/Rps2/Npm1/Prkca/Ppp1ca/Rpl10a/Rpl18/Rpl19/Rpl21/Rpl22/Rpl26/Rpl27/Rpl28/Rpl29/Rpl30/Rpl32/Rpl37a/Rpl36a/Rpl6/Rpl7/Rpl9/Rps12/Rps14/Rps15/Rps16/Rps17/Rps18/Rps19/Rps24/Rps29/Rps3a1/Rps4x/Rps5/Rps6/Rps7/Rps8/Eif1/Uba52/Ybx1/Rpl27a/Rps15a/Rpl23a/Rpl8/Rp
GO:0043043	M	peptide biosynthetic process	0.000999001	Rpl5/Rpl10/Rpl31/App/Rplp0/C1qbp/Eef1a1/Eif4a1/Fau/Rack1/Rpsa/Rps2/Npm1/Prkca/Ppp1ca/Rpl10a/Rpl18/Rpl19/Rpl21/Rpl22/Rpl26/Rpl27/Rpl28/Rpl29/Rpl30/Rpl32/Rpl37a/Rpl36a/Rpl6/Rpl7/Rpl9/Rps12/Rps14/Rps15/Rps16/Rps
GO:0043604	M	amide biosynthetic process	0.000999001	App/C1qbp/Cers6/Eef1a1/Eef1b2/Eef1d/Eef1g/Eif1/Eif3f/Eif3i/Eif3k/Eif4a1/Eif5a/Fau/Mrpl52/Ndufa7/Npm1/Prkca/Ppp1ca/Rack1/Rpl10/Rpl10a/Rpl1
GO:0006518	M	peptide metabolic process	0.000999001	Rpl5/Rpl10/Rpl31/Apoe/App/Rplp0/C1qbp/Eef1a1/Eif4a1/Fau/Rack1/Rpsa/Rps2/Npm1/Prkca/Ppp1ca/Rpl10a/Rpl18/Rpl19/Rpl21/Rpl22/Rpl26/Rpl27/Rpl28/Rpl29/Rpl30/Rpl32/Rpl37a/Rpl36a/Rpl6/Rpl7
GO:0043603	M	amide metabolic process	0.000999001	Rpl5/Rpl10/Rpl31/Apoe/App/Rplp0/C1qbp/Eef1a1/Eif4a1/Fau/Rack1/Rpsa/Rps2/Npm1/Prkca/Ppp1ca/Rpl10a/Rpl18/Rpl19/Rpl21/Rpl22/Rpl26/Rpl27/Rpl28/Rpl29/Rpl30/Rpl32/Rpl37a/Rpl36a/Rpl6/Rpl7/Rpl9/Rps12/Rps14/Rps15/Rps16/Rps17/Rps18/Rps19/Rps24/Rps29/Rps3a1/Rps4x/Rps5/

GO:1901566	M	organonitrogen compound biosynthetic process	0.000999001	Rpl5/Mgat5/Rpl10/Rpl31/Adk/Apoe/App/Rplp0/C1qbp/Ccnd3/Eef1a1/Eif4a1/Fau/Rack1/Rpsa/Rps2/Myc/Ndufa2/Nme1/Nme2/Npm1/Oaz1/Prkca/Ppp1ca/Rpl10a/Rpl18/Rpl19/Rpl21/Rpl22/Rpl26/Rpl27/Rpl28/Rpl29/Rpl30/Rpl32/Rpl37a/Rpl36a/Rpl6/Rpl7/Rpl9/Rps12/Rps14/Rps15/Rps16/Rps17/Rps18/Rps19/Rps24/Rps29/Rps3a1/Rps4x/Rps5/Rps6/Rps7/Rps8/Sat1/Sgms1/Eif1/Uba52/Vim/Zdhhc14/Ybx1/Impdh2/Nt5
GO:0009617	M	response to bacterium	0.000999001	Cr2/Ighg2b/Ighg1/Jchain/Mif/Slpi/Xbp1/Plac8/Igha/Scimp/Ighg3/Ighg2c
GO:0006396	M	RNA processing	0.000999001	Rpl5/Snrpd2/C1qbp/Hspa8/Npm1/Rpl10a/Rpl26/Rpl27/Rpl7/Rps16/Rps17/Rps19/Rps24/Rps6/Rps7/Rps8/Snrpe/Ybx1/Hnrnpa3/Rpl7a/Rps26/Rps28/Rpl35a/Ubl5/Nop10/Ahnak/Rps21/Rpl35/Rpl11/Rpl14/Snrpg/Rtraf/Rps13/Snrpf
GO:0046483	DM	heterocycle metabolic process	0.007992008	Rpl5/Rexo2/Eaf2/Snrpd2/Sri/Dut/Rpl10/Adk/Aldoa/Apex1/Birc5/Apoe/App/Aprt/Bach2/Phb2/Bmp6/Bmpr1a/C1qbp/Cacybp/Anxa2/Cbfa2t3/Ccnd3/Cct2/Cct3/Cct4/Cct5/Cct6a/Cct7/Cct8/Cxcr3/S1pr1/Eno1/Chchd2/Fbl/Fhit/Fli1/Tsc22d3/Hif1a/Hint1/Hmgb1/Hmgn2/
GO:0044085	M	cellular component biogenesis	0.003996004	Rpl5/Dnm3/Snrpd2/Rpl10/Actb/Actg1/Apoe/App/Rplp0/C1qbp/Cfl1/Coro1a/Cox17/Cox7a2/Dnah8/Fas/Fau/Gdi2/Rack1/Grb2/H2-Eb1/Hspa8/Jchain/Itgb1/Rpsa/Mif/Ndufa2/Npm1/Pfn1/Pip4k2a/Prkca/Tmsb10/Tmsb4x/Ptpn22/Ptprj/Rac2/Ran/Rpl10a/Rpl26/Rpl27/Rpl6/
GO:0098542	DM	defense response to other organism	0.008991009	Actg1/Apoe/Cr2/Cd55/Fcer1g/Hspa8/Hspd1/Ighg2b/Ighm/Jchain/Mif/Prdx1/Scimp/Ighg3
GO:0006139	DM	nucleobase-containing compound metabolic process	0.00999001	Rpl5/Rexo2/Eaf2/Snrpd2/Sri/Dut/Rpl10/Adk/Aldoa/Apex1/Birc5/Apoe/App/Aprt/Bach2/Phb2/Bmp6/Bmpr1a/C1qbp/Cacybp/Anxa2/Cbfa2t3/Ccnd3/Cct2/Cct3/Cct4/Cct5/Cct6a/Cct7/Cct8/Cxcr3/S1pr1/Eno1/Chchd2/Fbl/Fhit/Fli1/Tsc22d3/Hif1a/Hint1/Hmgb1/Hmgn2/Hnrnpa1/Hnrnpab/Hnrnpk/Prmt1/Hspa8/Hspd1/Hsp90aa1/Klf2/Aff3/Ldha/Sumo2/Magoh/Mdh2/Cd200/Myc/Naca/Ncl/Ndufa2/Nedd4/Nedd8/Nme1/Nme2/Npm1/Pfn1/Pa2g4/Raly/Ran/Rbm3/Rpl26/Rpl27/Rpl7/Sub1/Rps14/Rp

GO:0002252	DM	immune effector process	0.010989011	Iglc2/Cd80/Cr2/Cd55/Fcer1g/Fgl2/Hmgb1/Hspd1/Ighg2b/Ighm/Il9r/Lgals1/Mif/Prdx1/Ptprj/Rps6/Vpreb3/Xbp1/Scimp/Ighg3/Ighd
GO:0006725	DM	cellular aromatic compound metabolic process	0.013986014	Rpl5/Rexo2/Eaf2/Snrpd2/Sri/Dut/Rpl10/Adk/Aldoa/Apex1/Birc5/Apoe/App/Aprt/Bach2/Phb2/Bmp6/Bmpr1a/C1qbp/Cacybp/Anxa2/Cbfa2t3/Ccnd3/Cct2/Cct3/Cct4/Cct5/Cct6a/Cct7/Cct8/Cxcr3/S1pr1/Eno1/Chchd2/Fbl/Fhit/Fli1/Tsc22d3/Hif1a/Hint
GO:0051247	DM	positive regulation of protein metabolic process	0.016983017	Birc5/Apoe/App/Bmp6/C1qbp/Anxa2/Cd44/Cd80/Cdk4/Cfl1/Cycs/Eno1/Fas/Rack1/Hmgb1/Prmt1/Hspd1/Hsp90ab1/Ighm/Itgb1/Klf2/Laptm5/Sumo2/Marcks/Mif/Nedd9/Npm1/Psme2/Ptpn1/Ptprj/Rbm3/Rpl26/S100a10/Txn1/Xbp1/Ybx1
GO:1901360	DM	organic cyclic compound metabolic process	0.023976024	Rpl5/Rexo2/Eaf2/Snrpd2/Sri/Dut/Rpl10/Adk/Aldoa/Apex1/Birc5/Apoe/App/Aprt/Bach2/Phb2/Bmp6/Bmpr1a/C1qbp/Cacybp/Anxa2/Cbfa2t3/Ccnd3/Cct2/Cct3/Cct4/Cct5/Cct6a/Cct7/Cct8/Cxcr3/S1pr1/Eno1/Chchd2/Fbl/Fhit/Fli1/Tsc22d3/Hif1a/Hint1/Hmgb1/Hmgn2/Hn
GO:0002252	M	immune effector process	0.00999001	Foxp1/Iglc1/Iglc2/Iglc3/App/C1qbp/Cd2/Cd22/Cd80/Coro1a/Cr2/Ctsh/Cd55/Fas/Fgl2/Gpi1/H2-Ab1/Ighg2b/Ighg1/Il9r/Itgam/Itgb2/Laptm5/Lgals1/Cd180/Mif/Myo1f/
GO:0051050	DM	positive regulation of transport	0.027972028	Dnm3/Slc25a4/Apoe/App/Bmp6/Anxa2/Cfl1/Fcer1g/Hsp90ab1/Ighg2b/Ighm/Itgb1/Mif/Ndufa4/Edem1/Ptprj/Ran/S100a10/Xbp1/Ppia/Fbxw7/Park7/Tomm7/Anp32b
GO:0044085	DM	cellular component biogenesis	0.02997003	Rpl5/Arhgef18/Dnm3/Snrpd2/Dut/Rpl10/Actg1/Birc5/Apoe/App/C1qbp/Cct2/Cfl1/Cox17/Cox7a2/Dnah8/Fscn1/Fas/Fau/Fbl/Gas7/Gdi2/Rack1/H2-Eb1/Hmgb1/Prmt1/Hspa8/Hsp90aa1/Jchain/Itg
GO:0042325	DM	regulation of phosphorylation	0.035964036	Birc5/Apoe/App/Bmp6/Anxa2/Cd44/Cd80/Fas/Rack1/Hmgb1/Hsp90ab1/Ighm/Il9r/Itgb1/Marcks/Mif/Ncl/Nedd9/Npm1/Ppp1r14b/Ptpn1/Ptprj/Atxn1/Txn1/Xbp1/Pde4d/Pebp1/Zeb2/Ppia/Wdf
GO:0098542	M	defense response to other organism	0.015984016	Actg1/Apoe/Cr2/Ighg2b/Ighg1/Jchain/Mif/Slpi/Plac8/Igha/Scimp/Ighg3/Ighg2c

GO:0001932	DM	regulation of protein phosphorylation	0.044955045	Birc5/Apoe/App/Bmp6/Anxa2/Cd44/Cd80/Fas/Rack1/Hmgb1/Hsp90ab1/Ighm/Il9r/Itgb1/Marcks/Mif/Ncl/Nedd9/Npm1/Ptpn1/Ptprj/Txn1/Xbp1/Pde4d/Pebp1/Zeb2/Ppia/Wdfy2/Rps3/
GO:0008284	DM	positive regulation of cell population proliferation	0.043956044	Dnm3/Adk/Slc25a5/Apoe/Bmp6/Anxa2/Cd80/Cdk4/Cfl1/Cxcr3/Cd55/Hmgb1/Prmt1/Ighm/Itgb1/Marcks/Mif/Naca/Nedd9/Nme1/Nme2/Npm1/Pfn1/Ranbp1/Rps6/Rsu1/Atxn1/St6gal1/Xbp1/Pde4d/Zeb2/Rps3/Myo3b/Ighd/Plxnc1/Anp32b/Rpl4/Carmil1/Mzb1/Rps9

DM: DHA-treated mice; M: control mice
