# Supplementary material for: Dihydroartemisinin Alleviates the Symptoms of a Mouse Model of Systemic Lupus Erythematosus Through Regulating Splenic T/B-Cell Heterogeneity
Source: Curr Issues Mol Biol. 2025 Jul 9;47(7):528. doi: 10.3390/cimb47070528 (PMC12293267; doi:10.3390/cimb47070528)
Supplement: Supplementary file 1 [file cimb-47-00528-s001.zip › supplementary tables and figures/Table S8.pdf]

Table S8 Interaction counts of ligand and receptor in DM and M

Group	Source	Naive CD4_T	Effector CD4_T	Naive CD8_T	Effector CD8_T	Memory_T	Gamma delta_T	Follicular_B	Marginal Zone_B	Memory_B	Plasma
M	Naive CD4_T	8	9	22	27	9	11	12	11	18	2
M	Effector CD4_T	5	6	21	24	8	12	11	10	14	1
M	Naive CD8_T	7	10	22	27	10	11	9	7	15	1
M	Effector CD8_T	10	11	29	31	13	14	11	11	17	1
M	Memory_T	6	9	21	23	9	9	9	8	13	1
M	Gamma delta_T	11	11	25	27	10	14	8	8	12	0
M	Follicular_B	21	28	26	41	16	14	11	12	17	2
M	Marginal Zone_B	21	28	28	44	16	18	13	14	19	2
M	Memory_B	26	30	29	41	16	15	12	13	17	2
M	Plasma	3	3	10	14	0	2	2	2	1	0
DM	Naive CD4_T	6	8	20	25	5	8	11	11	11	1
DM	Effector CD4_T	3	6	20	23	7	14	9	10	9	1
DM	Naive CD8_T	3	10	23	23	9	8	8	7	9	1
DM	Effector CD8_T	7	11	28	30	11	12	8	8	11	1
DM	Memory_T	4	8	22	23	9	8	6	7	10	1
DM	Gamma delta_T	7	12	24	27	8	15	6	11	12	0
DM	Follicular_B	18	28	26	41	18	16	9	14	12	3
DM	Marginal Zone_B	15	27	23	38	17	16	9	10	10	2
DM	Memory_B	18	30	27	42	19	16	11	14	15	2
DM	Plasma	5	5	12	17	1	4	2	3	1	0
