# Supplementary material for: Dihydroartemisinin Alleviates the Symptoms of a Mouse Model of Systemic Lupus Erythematosus Through Regulating Splenic T/B-Cell Heterogeneity
Source: Curr Issues Mol Biol. 2025 Jul 9;47(7):528. doi: 10.3390/cimb47070528 (PMC12293267; doi:10.3390/cimb47070528)
Supplement: Supplementary file 1 [file cimb-47-00528-s001.zip › supplementary tables and figures/Table S9.pdf]

Table S9 Signaling values of ligand and receptor in DM and M

Group	Signaling pathway	Naive CD4_T	Other effector CD4_T	Naive CD8_T	Effector CD8_T	Memory_T	Gamma delta_T	Follicular_B	Marginal Zone_B	Memory_B	Plasma
DM	THY1	NA	NA	NA	NA	NA	NA	NA	NA	NA	NA
DM	MIF	0.10628734	0.10891299	0.126726546	0.103892335	0.094164086	0.087878739	0.102747486	0.210988463	1	0.001327408
DM	TGFb	NA	0.150023557	0.557464332	NA	0.622148974	NA	0.533562542	0.69219225	1	NA
DM	PECAM1	0.794574058	0.22505314	NA	NA	NA	NA	0.99927285	1	0.754692645	NA
M	THY1	0.149629311	0.179972991	0.264456259	0.259418458	0.146522982	NA	NA	NA	1	NA
M	MIF	0.142778752	0.149285512	0.107983753	0.150374839	0.135203435	0.107874188	0.422235411	0.321178908	1	NA
M	TGFb	NA	0.366549955	0.662034717	0.366515123	0.651380167	0.110800329	1	0.945164203	0.93744511	NA
M	PECAM1	0.455392996	0.372009691	NA	NA	NA	NA	0.98391763	1	0.840517876	NA
